# TNF-α Blocker Effect of Naringenin-Loaded Sericin Microparticles that Are Potentially Useful in the Treatment of Psoriasis

**DOI:** 10.3390/ijms150813624

**Published:** 2014-08-06

**Authors:** Theodora Chlapanidas, Sara Perteghella, Flavio Leoni, Silvio Faragò, Mario Marazzi, Daniela Rossi, Emanuela Martino, Raffaella Gaggeri, Simona Collina

**Affiliations:** 1 Department of Drug Sciences, Medicinal Chemistry and Pharmaceutical Technology Section (MCPTS), Cell Delivery System Laboratory, University of Pavia, Viale Taramelli 12, 27100 Pavia, Italy; E-Mails: theodora.chlapanidas@unipv.it (T.C.); sara.perteghella@unipv.it (S.P.); 2 Italfarmaco Research Center, Via dei Lavoratori 54, 20092 Cinisello Balsamo, Italy; E-Mail: f.leoni@italfarmaco.com; 3 Innovhub, Stazioni Sperimentali per l’Industria, Divisione Seta, Via Giuseppe Colombo 83, 20133 Milan, Italy; E-Mail: silvio.farago@mi.camcom.it; 4 Struttura Semplice Tissue Therapy, Niguarda Hospital, Piazza dell’Ospedale Maggiore 3, 20162 Milan, Italy; E-Mail: mario.marazzi@ospedaleniguarda.it; 5 Department of Drug Sciences, Medicinal Chemistry and Pharmaceutical Technology Section (MCPTS), Medicinal Chemistry Laboratory, University of Pavia, Viale Taramelli 12, 27100 Pavia, Italy; E-Mails: daniela.rossi@unipv.it (D.R.); raffaella.gaggeri@unipv.it (R.G.); 6 Department of Earth and Environmental Sciences, University of Pavia, Via S. Epifanio 14, 27100 Pavia, Italy; E-Mail: emanuela.martino@unipv.it; 7 Center for Studies and Researches in Ethnopharmacy (C.I.St.R.E.), University of Pavia, Viale Taramelli 12, 27100 Pavia, Italy

**Keywords:** flavanones, Naringenin, sericin, microparticles, psoriasis, anti-inflammatory activity, TNF-α

## Abstract

This study aims to evaluate the effect of combined use of the racemic flavanone Naringenin (NRG) and the protein sericin as TNF-α blockers. Sericin (SMs) and (*R*/*S*) NRG-loaded Sericin (SNRGMs) microparticles were prepared by spray-drying, characterized in terms of morphology and particle size distribution, and encapsulation efficiency was determined. Concerning morphology and particle size distribution of microparticles, results indicated that they were not affected by the presence of NRG. The encapsulation efficiency was almost quantitative (93%), thus proving that sericin can be advantageously loaded with (*R*/*S*) NRG. Biological evaluation of (*R*/*S*) NRG, SMs and SNRGMs was then performed in lipopolysaccharide (LPS)-stimulated human peripheral blood mononuclear cells (hPBMC). SNRGMs resulted cytotoxic at the higher dose used (200 μg/mL) and the effect was greater than (*R*/*S*) NRG alone. Moreover, even if sericin alone was not effective in suppressing LPS-induced serum TNF-α levels, SNRGMs loaded with 9.3% of (*R*/*S*) NRG were significantly more potent than (*R*/*S*) NRG alone. In summary, this study provides the proof of concept that sericin-based microspheres loaded with TNF-α-blockers could contribute to the down regulation of the cytokine and represents the starting point for the development of new topical formulations for the treatment of middle-stage psoriasis.

## 1. Introduction

**Figure 1:**
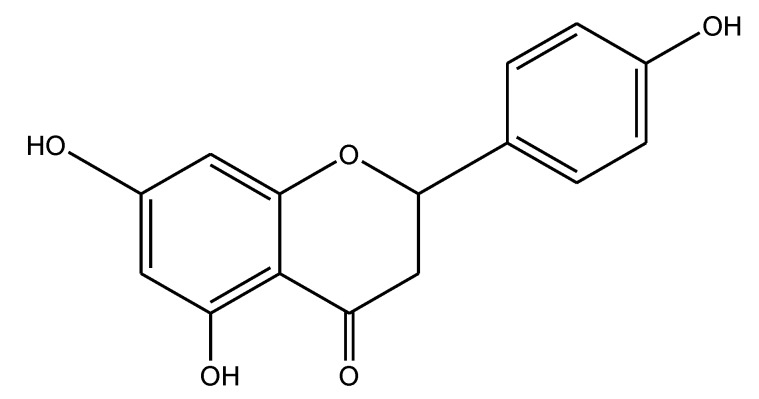
Chemical structure of (*R*/*S*) Naringenin (NRG).

Our ethnobotanical-directed search for novel anti-inflammatory drugs revealed that *Amygdalus lycioides* Spach, an Iranian medicinal plant, holds interesting anti-inflammatory activity exerted via a TNF-α-blocker mechanism [1,2]. Following the bio-guided fractionation approach, we demonstrated that the (*S*) enantiomer of Naringenin (4',5,7-trihydroxyflavanone, NRG, Figure 1) is the metabolite responsible for the observed TNF-α blocker effect [1,2]. Further studies, carried out to compare the TNF-α inhibitory activity of racemic NRG and its pure enantiomers, clearly demonstrated that both NRG enantiomers are effective in inhibiting TNF-α production in a dose-dependent manner [3]. Subsequently, we also studied the anti-inflammatory properties of silk sericin. It is a protein widely used in pharmaceutical, medical and cosmetic fields because of its advantageous properties such as biocompatibility, biodegradability, gelling ability and skin adhesion [4]. In detail, we proposed the use of sericin in inflammatory/autoimmune diseases like psoriasis since it appeared to be active in an *in vitro* model of T-cell activation [5]. In particular, sericin was shown to inhibit cell proliferation of human peripheral blood mononuclear cells (hPBMC) stimulated by the mitogen phytohaemagglutinin (PHA) [5]. These results suggested that we evaluate the effect of the combined use of (*R*/*S*) NRG and sericin as TNF-α blockers. Thus, we addressed our efforts towards the loading of (*R*/*S*) NRG into sericin microparticles, followed by the evaluation of their inhibitory effect in a cellular model of TNF-α production (*i.e.*, lipopolysaccharide (LPS)-stimulated hPBMC). We hoped to develop new topical formulations potentially useful in the treatment of psoriasis. Indeed, recent genetic and immunological advances have greatly increased understanding of the pathogenesis of this disease as an immune-mediated inflammatory disorder [6], in which the cytokine TNF-α plays a crucial role [7,8].

## 2. Results

### 2.1. Preparation, Characterization and Analysis of Naringenin-Loaded Sericin Microspheres

**Figure 2:**
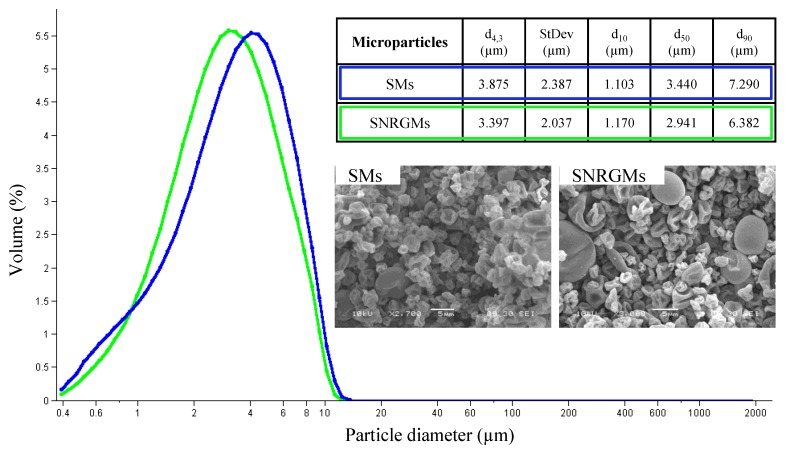
Particle size distribution of SMs (blue line) and SNRGMs (green line). The inner table reports the mean volume-weighted diameter (*d*_4,3_), the *d*_4,3_ standard deviation (SD), the 10th, the 50th and the 90th percentile of *d*_4,3_ (*d*_10_, *d*_50_ and *d*_90_, respectively). The SEM images are reported for both types of microparticles. Scale Bar: 5 μm.

At first, we prepared Sericin (SMs) and (*R*/*S*) NRG-loaded Sericin (SNRGMs) microspheres by a spray drying method. As evidenced by laser light scattering analysis (Figure 2), the dimensions of both microspheres were similar and the presence of NRG did not influence the particle size distribution. Particularly, both microparticles presented an unimodal distribution with a mean volume-weighted diameter (*d*_4,3_) of 3.875 ± 2.387 and 3.397 ± 2.037 µm, for SMs and SNRGMs respectively (Figure 2). Moreover, the scanning electron microscopy analysis (Figure 2) indicated that both types of microparticles were collapsed and presented a rough surface.

(*R*/*S*) NRG was quantified by means of HPLC-UV/PAD (high performance liquid chromatography-ultraviolet photodiode array detection) analysis using a Chromolit Speed-ROD RP18 and eluting with water containing 0.1% (*v*/*v*) formic acid (A) and methanol (B) in gradient conditions [2]. The selectivity of the method was guaranteed by the photodiode array detector. Indeed, in the chromatogram of SMs no peak co-eluted with NRG was observed, thus ensuring that sericin does not interfere with (*R*/*S*) NRG analysis. The method repeatability was assessed by performing three injections on different days. Quantitative (*R*/*S*) NRG determination in SNRGMs was performed using external standards by means of five-point calibration curves. The method presented a good linearity in the concentration range of 5–0.3125 mg/mL and the calibration curve (*y* = (6 × 10^−8^)*x* − 0.0285) showed a good correlation coefficient (*R* = 0.9987).

To develop a procedure suitable for (*R*/*S*) NRG recovery calculation, the different solubility of (*R*/*S*) NRG and sericin in methanol was exploited. Briefly, the methanolic suspension containing a known amount of (*R*/*S*) NRG with SMs (sample named NRG + SMs) was sonicated and (*R*/*S*) NRG quantified at different time points by HPLC analysis. After 4 h of sonication a recovery of 95% was obtained.

Concerning the encapsulation efficiency (EE of (*R*/*S*) NRG in SNRGMs), *i.e.*, the amount of added analytes that is encapsulated in the formulation of microparticles, it was calculated in terms of the ratio of (*R*/*S*) NRG amount present in the final formulation (SNRGMs) and the amount of (*R*/*S*) NRG added during the encapsulation process. HPLC analysis of three different batches of SNRGMs evidenced that the (*R*/*S*) NRG amount was 9.3% ± 0.4%, and consequently, the encapsulation efficiency obtained was 93%.

### 2.2. Biological Evaluation

**Figure 3:**
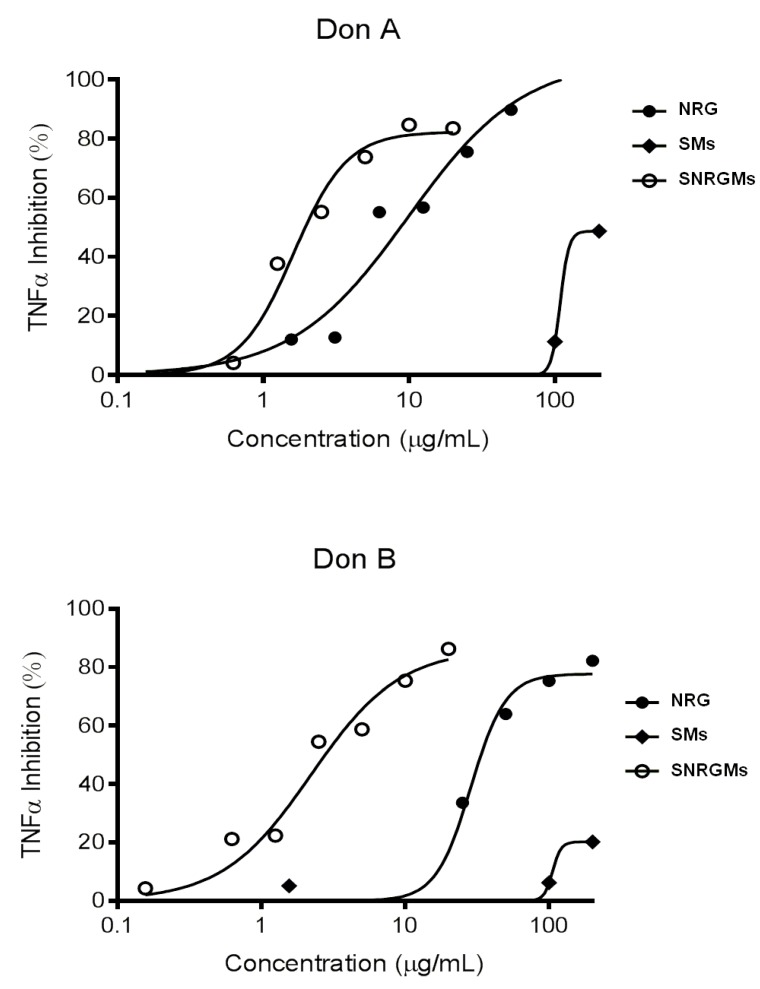
Inhibitory dose-response effect of (*R*/*S*) NRG, SMs and SNRGMs on TNF-α production from LPS-stimulated hPBMC from two different normal donors.

We firstly defined a range of doses devoid of nonspecific cytotoxic effects, according to the experimental approach already applied in our study on (*R*/*S*) NRG [3]. To this aim, hPBMC were stimulated for 24 h with 10 ng/mL of LPS in the presence of serial dilutions (from 3.1 to 200 μg/mL) of (*R*/*S*) NRG, SMs and SNRGMs and then the cell viability was evaluated by Alamar assay. Results reported in Table 1 clearly showed that: (i) SMs have a negligible (<15% inhibition) cytotoxic activity at all the used doses on hPBMC of both donors or, even, a slight stimulation (20.6%) of cell viability at the higher dose on Donor A; (ii) in accordance to previous findings, (*R*/*S*) NRG significantly inhibits cell viability only at the higher dose (200 μg/mL) on both donors (26.6% and 23.7% inhibition); and (iii) SNRGMs is cytotoxic at the higher dose used and the effect is greater than that of NRG (73.1% and 67.2% inhibition on Donor A and Donor B, respectively). The non cytotoxic working concentrations of (*R*/*S*) NRG, SMs and SNRGMs were used in the following test in order to avoid possible interference with cell viability. Therefore, we evaluated the *in vitro* TNF-α inhibitory activity of (*R*/*S*) NRG, SMs and SNRGMs, using hPBMC from two different normal donors treated with LPS as inflammatory stimulus. After 24 h, the resulting TNF-α concentration was determined using commercial ELISA. The results reported in Figure 3 indicate that: (i) SMs is slightly active at the higher dose used (200 µg/mL) in inhibiting TNF-α production on Donor B and partly active (about 40% inhibition) at the higher dose used on Donor A; (ii) as already evidenced, (*R*/*S*) NRG alone inhibits TNF-α production on both donors in a dose-dependently manner; and (iii) SNRGMs seem to be more active than the free NRG since its dose-response curve is shifted towards lower concentrations. This pattern of activity becomes more evident if we compare the potencies of the three compounds in terms of IC_50_ (Table 2).

In detail, the IC_50_ values of SMs are about 200 µg/mL on Donor A and higher than 200 µg/mL on Donor B (about 20% TNF-α inhibition at 200 µg/mL), confirming that SMs alone are not effective in inhibiting TNF-α production. In contrast, (*R*/*S*) NRG showed an interesting activity with an IC_50_ of 8.7 μg/mL on Donor A and 33.6 μg/mL on Donor B and, above all, SNRGMs is significantly more potent than (*R*/*S*) NRG alone having a IC_50_ of 1.9 µg/mL (Donor A) and of 2.8 μg/mL (Donor B) with a 4.6- and 12.0-fold increased potency, respectively.

**Table 1:** Effect of SMs, (*R*/*S*) NRG and SNRGMs on cell viability (Alamar assay) of LPS-stimulated hPBMC from two different normal donors. The mean of fluorescence (expressed as arbitrary fluorescence units, AFU) and standard deviation (SD) are also reported in parentheses.

Concentration (µg/mL)	Percent Inhibition of Cell Viability (AFU Fluorescence ± SD)					
	Donor A			Donor B		
	SMs	NRG	SNRGMs	SMs	NRG	SNRGMs
0.0	(216,176 ± 16,662)	(216,176 ± 16,662)	(216,176 ± 16,662)	(296,090 ± 3485)	(296,090 ± 3485)	(296,090 ± 3485)
1.5	−0.6 (217,369 ± 23,816)	12.7 (188,915 ± 5021)	12.0 (190,284 ± 17,929)	5.7 (279,202 ± 19,030)	7.3 (274,536 ± 15,953)	2.8 (287,884 ± 9992)
3.1	3.4 (208,859 ± 12,809)	7.7 (199,427 ± 12,120)	13.5 (186,912 ± 19,949)	−0.5 (297,628 ± 2021)	3.9 (284,657 ± 10,232)	3.8 (284,955 ± 15,984)
6.2	−1.7 (219,873 ± 3647)	4.9 (205,540 ± 2716)	9.1 (196,516 ± 7684)	−0.2 (296,641 ± 8079)	4.0 (284,141 ± 8173)	−1.3 (299,915 ± 5812)
12.5	6.6 (201,875 ± 23,690)	−7.4 (232,158 ± 8719)	−3.6 (224,022 ± 8827)	12.0 (260,699 ± 21,230) *	2.4 (288,952 ± 12,039)	−5.6 (312,652 ± 817)
25	14.4 (185,018 ± 8764)	−8.1 (233,665 ± 14,492)	−17.5 (254,054 ± 5993) *	8.4 (271,324 ± 22,478)	−2.1 (302,298 ± 5691)	−6.8 (316,310 ± 15,836)
50	−0.4 (216,983 ± 18,889)	0.4 (215,207 ± 10,787)	−11.0 (239,937 ± 17,440)	4.0 (284,183 ± 20,694)	−4.5 (309,485 ± 6492)	−10.2 (326,391 ± 9586)
100	−2.9 (222,453 ± 17,007)	9.1 (196,415 ± 9256)	−18.6 (256,290 ± 6843)	−2.4 (303,159 ± 29,542)	4.1 (284,065 ± 10,582)	−12.1 (331,907 ± 6113) *
200	−20.6 (260,790 ± 23,226) **	26.6 (158,719 ± 12,585) ***	73.1 (58,242 ± 7469) ***	−3.3 (305,767 ± 23,492)	23.7 (225,848 ± 12,064) ***	67.2 (97,067 ± 27,795) ***

* *p* < 0.05, ** *p* < 0.01, *** *p* < 0.001 by 2-way ANOVA followed by multiple comparison test.

**Table 2:** TNF-α inhibitory activity (IC_50_) of SMs, (*R*/*S*) NRG and SNRGMs on LPS-stimulated hPBMC from two different normal donors.

Treatment	IC_50_ (μg/mL)	
	Donor A	Donor B
SMs	200 *	>200 **
NRG	8.7	33.6
SNRGMs ***	1.9	2.8
Fold increase	4.6	12.0

* 48.7% inhibition at 200 μg/mL; ** 20.2% inhibition at 200 μg/mL; *** IC_50_ referred to NRG contained in SNRGMs.

## 3. Discussion

Treatments available for psoriasis have increased rapidly in recent years; however, they are still incomplete. Thanks to the discovery of new immunological factors and also to a better understanding of the functioning of psoriasis, the research community has turned its focus on immunological pathways and addressed its efforts towards the development of new drugs targeting pathways involved in the etiology of this pathology [7,9]. In this context, anti-TNF-α drugs (*i.e*., Infliximab, Etanercept and Adalimumab) have been developed to capture the TNF-α and to block its activity and consequently to reduce the interactions between immune cells and keratinocytes [10,11]. However, these biological drugs are suitable only for systemic administration and, in addition, they are currently very expensive. For these reasons, the discovery of new drugs, acting via the inhibition of TNF-α production and suitable for topical administration is still an established need.

Recently, we demonstrated that (*R*/*S*) NRG and sericin could both be useful in inflammatory diseases treatment. Indeed, our previous studies clearly evidenced that NRG is endowed with a TNF-α blocking effect [2,3], while sericin has an inhibitory effect on PHA-activated hPBMC [5]. In the present study we have evaluated for the first time the effect of the combined use of (*R*/*S*) NRG and sericin on TNF-α production, using the *in vitro* LPS-induced inflammation model. To this aim, our strategy consisted of loading NRG in sericin microspheres and then evaluating the effect of NRG-loaded sericin microspheres on TNF-α production in our *in vitro* model. As discussed in the introduction section, the use of sericin in biomedical fields is widely studied [4], but sericin-based microspheres loaded with drugs were only recently proposed for drug delivery applications. Indeed, to the best of our knowledge, only one paper describing sericin microspheres loaded with an active principle (diclofenac was used as model drug) has been published so far [12]. In detail, in the present paper we prepared two different batches of microparticles: sericin and sericin microspheres loaded with (*R*/*S*) NRG, named SMs and SNRGMs respectively, in order to evaluate their effect in the down regulation of TNF-α. In both cases, microparticles were successfully prepared and, according to results previously obtained by our research group [5], the particle size distributions were in the range of 3.3–3.8 µm (*d*_4,3_) for both batches, thus suggesting that the presence of NRG did not influence the particle size distribution. The content of (*R*/*S*) NRG in SNRGMs was determined using an economic HPLC-UV/PAD method. Interestingly, the encapsulation efficiency observed was almost quantitative (93%), thus proving that sericin can be advantageously loaded with (*R*/*S*) NRG. After microspheres characterization, the biological investigation of SMs, (*R*/*S*) NRG and SNRGMs was carried out. The results indicated that (i) SMs did not have cytotoxic effect on hPBMC stimulated by LPS (an opposite trend was observed with PHA-stimulated cells [13]); (ii) (*R*/*S*) NRG significantly inhibited cell viability only at the higher dose (200 μg/mL); and (iii) SNRGMs are cytotoxic at the higher dose used. It should be noted that the (*R*/*S*) NRG content of SNRGMs is only 9.3%, hence 200 µg/mL of SNRGMs corresponded to 20 µg/mL of (*R*/*S*) NRG, a dose without toxic effect on both donors, in agreement with our previous investigations [3]. Regarding the ability of SMs, (*R*/*S*) NRG and SNRGMs to suppress LPS-induced serum TNF-α production, it is noteworthy that, even if sericin alone was not effective, SNRGMs loaded with 9.3% of (*R*/*S*) NRG were significantly more potent than NRG alone (Table 2). Indeed, (*R*/*S*) NRG showed an IC_50_ of 8.7 μg/mL on Donor A and 33.6 μg/mL on Donor B, while SNRGMs showed IC_50_ values of 1.9 µg/mL (Donor A) and of 2.8 μg/mL (Donor B).

Taken together, results of cytotoxicity and of LPS-induced TNF-α levels inhibition studies clearly evidenced that when NRG is loaded into sericin microspheres is more effective than NRG alone.

## 4. Experimental Section

### 4.1. Materials

**Figure 4:**
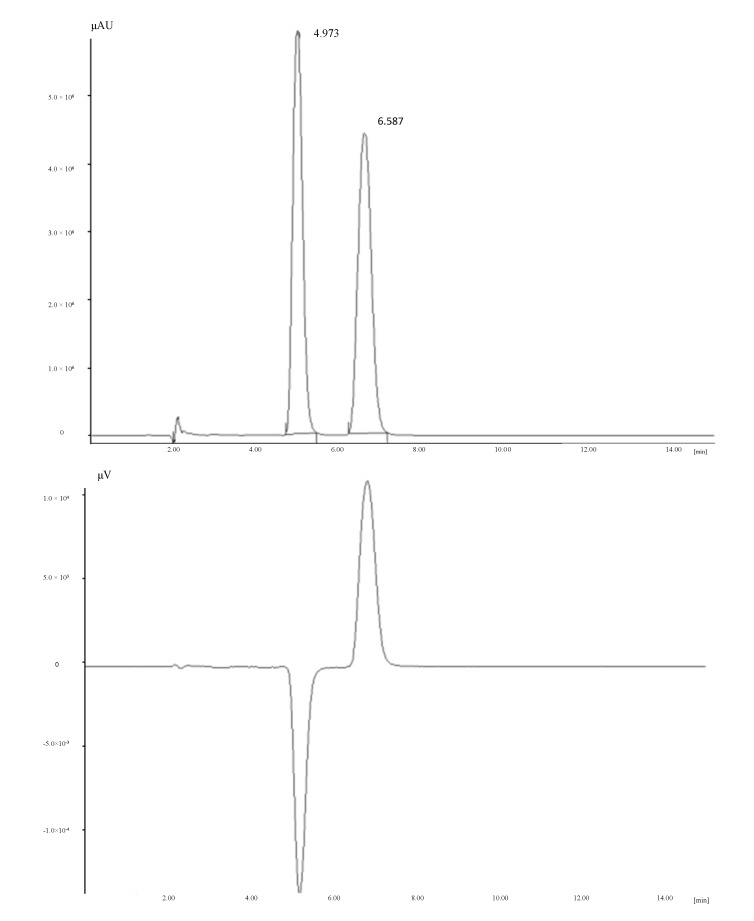
UV (**upper**) and circular dichroism (CD) (**lower**) chromatograms (recorded at 290 nm) of (*R*/*S*)-NRG on Chiralpak AD-H (150 × 4.6 mm I.D., *d_p_* = 5 µm), eluent: 100% methanol, flow: 1 mL/min.

(*R*/*S*)-NRG (5,7-dihydroxy-2-(4-hydroxyphenyl)chroman-4-one) was obtained from Sigma–Aldrich (Milan, Italy). To check the NRG composition in terms of proportion of *R* and *S* enantiomers, enantioselective HPLC analysis was performed on a Jasco system (JASCO Europe, Cremella, Italy) consisting of a PU-2089 plus pump, a AS-2055 plus autosampler, a MD-2010 plus detector and a CD-2095 plus circular dichroism (CD) detector, using a Chiralpak AD-H column (150 × 4.6 mm I.D., *d_p_* = 5 µm) and eluting with 100% methanol at a flow rate of 1 mL/min (Figure 4).

All solvents used as eluents were HPLC grade and were obtained from Carlo Erba (Milan, Italy).

### 4.2. Preparation of Sericin and Naringenin-Loaded Sericin Microspheres

Sericin extraction was performed as previous reported [5]. Briefly, *Bombyx mori* cocoons were put in autoclave at 120 °C for 1 h (40 mL of water/g of cocoon). Sericin solution was dried using a Büchi mini spray dryer (pump, 6 mL/min; inlet temperature, 120 °C; outlet temperature, 80 °C; air pressure, 3 bar; fluid flow, 500–600 mL/h, Büchi, Cornaredo, Italy) in order to obtain sericin microspheres (SMs). SMs were divided in two aliquots: the first one was used as such for the further experiments, while the second one was processed in order to obtain NRG-loaded sericin microspheres (SNRGMs). In particular, SMs were dissolved in water, NRG was dissolved in ethanol and added to the sericin aqueous solution. The resulting suspension was composed by sericin 0.8% *w*/*v* and NRG 0.09% *w*/*v* and was dried by spray drying using process parameters above. The theoretical composition of SNRGMs was 90% sericin and 10% NRG. Each experiment was performed in triplicate. The morphology of SMs and SNRGMs was evaluated under a scanning electron microscope JEOL JSM-6380LV (JOEL, Basiglio, Italy), operating at low vacuum degree, 20 kV, retrodiffused electron signal.

### 4.3. Granulometric Analysis

Ethanol suspensions of microspheres (SMs and SNRGMs) were put into the measurement cell of laser light scattering granulometer (Beckman Coulter LS230, Beckman Coulter, Brea, CA, USA), equipped with a small volume cell (120 mL volume with refractive index set at 1359 for ethanol, obscuration 5%). Results are expressed as the mean value of the five replicates.

### 4.4. HPLC Analysis of NRG-Loaded Sericin Microspheres

(*R*/*S*) NRG was quantified by high performance liquid chromatography-ultraviolet photodiode array detection (HPLC-UV/PAD). A JASCO system (JASCO Europe, Cremella, Italy) equipped with a Jasco AS-2055 plus autosampler, a PU-2089 plus pump and a MD-2010 plus multiwavelength detector was used. Experimental data were acquired and processed by Borwin PDA and Borwin Chromatograph Software (JASCO Europe). Chromatographic separations were carried out using a Chromolith SpeedROD RP-18 endcapped column (50 × 4.6 mm, I.D. 3 mm, macropore size 2 μm, mesopore size 13 nm, Merck, KGaA, Darmstadt, Germany) and a security guard H5-10C column. The mobile phase consisted of water containing 0.1% (*v*/*v*) formic acid (A) and methanol (B) and elution was performed in gradient conditions (from 90% of A to 60% of A within 20 min, followed by a re-equilibration step of 5 min). The flow rate was set at 1 mL/min and detection was fixed at 290 nm. Injection volume was 5 µL. Quantitative determination of NRG in SNRGMs was performed using external standards by means of five-point calibration curve. A stock solution was prepared at 10 mg/mL dissolving standard (*R*/*S*) NRG in methanol at room temperature. The stock solution was then serially diluted in methanol by two folds to obtain standard calibration solutions, at concentrations of 5, 2.5, 1.25, 0.625, 0.3125 mg/mL. Each standard calibration solution was injected in triplicate. Method recovery was investigated by spiking 2 mL of NRG methanolic solution (5 mg/mL) to 100 mg of SMs (sample named NRG + SMs). The NRG + SMs suspension was accurately mixed and dried up under nitrogen flow till reached a constant weight. The dried sample was then suspended in 10 mL of pure methanol and sonicated at room temperature for 4 h. After that, the suspension was centrifuged at 3000 rpm for 10 min and the supernatant was filtered and injected into the HPLC system. The overall process was carried out in triplicate. The recovery was determined by comparing the peak area response of NRG + SMs sample to that of the NRG standard calibration solution having the same concentration (1 mg/mL), according to the following formula:

Recovery% = (Area_Found_/Area_STD_) × 100% (1)

where Area_Found_ is Area of NRG + SMs sample and Area_STD_ is the Area of NRG standard solution at 1 mg/mL.

### 4.5. Encapsulation Efficiency Determination

An accurately weighted amount (50 mg) of SNRGMs was suspended in 10 mL of methanol and sonicated for 4 h at room temperature; the suspension was centrifuged at 3000 rpm for 10 min and the supernatant filtered. The amount of NRG in SNRGMs was determined applying the HPLC method described above, taking into account the recovery percentage. The encapsulation efficiency (EE) of NRG in SNRGMs was calculated as follows:

EE% = ([NRG]_Found_/[NRG]_add_) × 100% (2)

where [NRG]_Found_ are mg of encapsulated NRG and [NRG]_add_ are mg of NRG added during the encapsulation process. The experiment was performed in triplicate.

### 4.6. Human Peripheral Blood Mononuclear Cells Preparation

Human peripheral blood mononuclear cells (hPBMC) were obtained by centrifugation over Ficoll–Hypaque cushions of buffy-coats from two human normal donors (Donor A and Donor B), as previously described [1,13], counted using a cell counter (AcT5diff Beckman Coulter, Beckman Coulter Italia, Cassina De’Pecchi, Italy) and re-suspended at 5 × 10^6^ cells/mL in tissue culture medium RPMI 1640 (Biochrom, distributed by BioSpa, Milan, Italy) containing 1% fetal calf serum (FCS) (Hyclone, distributed by Thermo Scientific, Rodano, Italy) and placed at +4 °C overnight. The next morning hPBMC were re-suspended and 100 µL added to triplicate wells of 96-well plates.

### 4.7. Cytotoxicity Assay

The assay was carried out according to manufaturer (Invitrogen, S. Giuliano Milanese, Italy) indications. Briefly, the cells were stimulated with *Escherichia coli* O55B5 lipopolysaccharide (LPS, Sigma–Aldrich) at the final concentration of 10 ng/mL for 1 h at 37 °C. The cells were then treated (in triplicate) for additional 23 h with serial concentrations of SMs, (*R*/*S*) NRG and SNRGMs (from 3.1 to 200 μg/mL, dilution factor = 3) in a final volume of 200 μL of RPMI 1640 and 1% FCS. Control untreated cells were treated with RPMI 1640 and 1% FCS. The Alamar blue dye (Invitrogen) was added for the last 4 h of treatment and the fluorescence (expressed as arbitrary fluorescence units, AFU) of the medium of each well measured (excitation 530–560 nm, emission 590 nm) by using a plate fluorometer (Victor Wallac Multilabel Counter 1420, Perkin–Elmer, Milan, Italy).

Cytotoxicity was expressed as percent inhibition of cell viability, as follows:

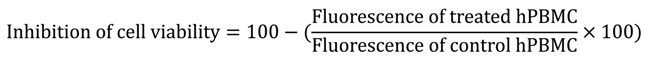
(3)


### 4.8. TNF-α Inhibition Assay

The assay was carried on separated plates prepared as for the cytotoxicity assay described above. At the end of the 24 h period of treatment 100 µL of the supernatant of each well were harvested and used to measure TNF-α using a commercial ELISA kit (R&D system, distributed by Space srl, Milan, Italy) for the human cytokine, according to manufacturer indications. The inhibition of TNF-α was expressed as IC_50_ (concentration reducing by 50% TNF-α level of treated cells in respect to control cells).

### 4.9. Statistical Analysis

IC_50_ of TNF-α inhibition was determined from the non-linear regression analysis of the dose-response curve by using GraphPad Prism 5.0 statistical software (GraphPad Software, Inc., La Jolla, CA, USA). Inhibition of cell viability data were analyzed by two-way ANOVA followed by multiple comparison test by using GraphPad Prism 5.0 statistical software. The statistical significance level was set at *p* < 0.05.

## 5. Conclusions

Overall results herein presented indicate that sericin microparticles loaded with naringenin are more potent than (*R*/*S*) NRG alone in inhibiting the TNF-α production on LPS-stimulated human PBMC and, also, that this effect is not due to an unspecific cytotoxic activity. Most importantly, our *in vitro* studies provide the proof of concept that sericin-based microspheres loaded with TNF-α-blocker molecules could contribute to the down regulation of the cytokine. Therefore, these results represent the starting point for the development of new topical formulations suitable for the treatment of middle-stage psoriasis, a pathology which requires the application of topical agents to a small area. Moreover, it has to be outlined that the plaques of psoriasis could benefit from the use of sericin. Indeed, thanks to its occlusive effect, sericin may improve skin barrier function, thus preventing water loss from the upper layer of the skin [14]. Current researches are addressed to better define the putative synergic activity of sericin microparticles and (*R*/*S*) NRG. Results of the biological investigation of sericin-based microparticles loaded with (*R*/*S*) NRG and the physical mixtures of these compounds will be reported in due course. In case of positive outcomes, our efforts will be directed towards the evaluation of potential *in vivo* or *ex vivo* efficacy in psoriasis.
